# Synthesis, crystal structure, DFT, Hirshfeld surface analysis, energy frameworks and in-Silico drug-targeting PFKFB3 kinase of novel triazolequinoxalin derivative (TZQ) as a therapeutic Strategy against cancer

**DOI:** 10.1016/j.heliyon.2023.e21312

**Published:** 2023-10-20

**Authors:** Nadeem Abad, Fares Hezam Al-Ostoot, Sajda Ashraf, Karim Chkirate, Majed S. Aljohani, Hussam Y. Alharbi, Shafeek Buhlak, Mohamed El Hafi, Luc Van Meervelt, Basheer M. Al-Maswari, El Mokhtar Essassi, Youssef Ramli

**Affiliations:** a Department of Biochemistry, Faculty of Education & Science, Al-Baydha University, Yemen; b Laboratory of Heterocyclic Organic Chemistry URAC 21, Pharmacochemistry Competence Center, Av. Ibn Battouta, BP 1014, Faculty of Sciences, Mohammed V University in Rabat, 10010, Morocco; c Dr.PanjwaniCenter for Molecular Medicine and Drug Research, International Center for Chemical and Biological Sciences, University of Karachi, Karachi 75270, Pakistan; d Department of Chemistry, Faculty of Science, Taibah University, Yanbu, Saudi Arabia; e Department of Chemistry, Abantİzzet Baysal University, 14280 Bolu, Turkey; f Laboratory of Biomolecular Architecture, Department of Chemistry, KU Leuven, Celestijnenlaan 200F, Leuven, B-3001, Belgium; g Department of Chemistry, Yuvaraja's College, University of Mysore, Mysuru, Karnataka 570005, India; h Laboratory of Medicinal Chemistry, Drug Sciences Research Center, Faculty of Medicine and Pharmacy, Mohammed V University in Rabat, Morocco

**Keywords:** Triazolequinoxalin, Energy framework, Hirshfeld surface, Anti-cancer docking studies

## Abstract

Overall, drug design is a dynamic and evolving field, with researchers constantly working to improve their understanding of molecular interactions, develop new computational methods, and explore innovative techniques for creating effective and safe medications. The process can involve steps such as the identification of targets, the discovery of lead compounds, lead optimization, preliminary testing, human trials, regulatory approval and finally post-marketing surveillance, all aimed at bringing a new drug from concept to market. In this article, the synthesis of the novel triazolequinoxalin (**TZQ**) 1-((1-hexyl-1H-1,2,3-triazol-5-yl)methyl)-3-phenylquinoxalin-2(1H)-one (**4**) is reported. The structure has been identified with a variety of spectroscopic methods (1H, 13C NMR, and LC-MS) and finally, the structure has been determined by X-ray diffraction (XRD) studies. The **TZQ** molecule has crystallized in the monoclinic space *C*2/*c* group with unit cell dimensions *a* = 41.201(2) Å, *b* = 10.6339(6) Å, *c* = 9.4997(4) Å, *β* = 93.904(4). The crystal structure is stabilized by intermolecular interactions (N–H ⋯ O and N–H … Cg) occurring within the molecule. The presence of these intermolecular interactions is evaluated through analysis of Hirshfeld surfaces (HS) and two-dimensional (2D) chemical fingerprints map. Additionally, energy frameworks were employed to identify the prevailing interaction energy influencing the molecular arrangement. Density Functional Theory (DFT) calculations were computed to establish concurrence between theoretical and experimental results. Furthermore, the HOMO-LUMO energy levels were determined using the B3LYP/6-31+G(d, p) level of theory. Finally, molecular docking was used to predict the anti-cancer activity of the compound (**4**) against PFKFB3 kinase and presented noticeable hydrophilic and hydrophobic interactions at the active site region.

## 1 Introduction

While there have been significant advancements in cancer treatment, many current drugs can cause serious side effects and may not be effective enough in certain cases [1]. The development of new, safer, and more effective drugs for cancer is crucial [[2], [3], [4]]. This requires a greater comprehension of the underlying mechanisms of cancer and the identification of new targets for therapy [5]. Additionally, research into personalized medicine, which tailors treatment to an individual's specific genetic and molecular makeup, holds great promise for improving cancer outcomes [6,7]. It's important to note that even with advanced-stage cancer, there is still hope for effective treatment and even remission in some cases [8]. With advancements in cancer research and treatment, patients with advanced-stage cancer are living longer and experiencing improved quality of life [9]. Additionally, palliative care and supportive therapies aim to enhance the comfort, well-being, and quality of life of patients with advanced cancer. Most recently, there have been substantial advancements in combine of chemotherapy and immunotherapy, which use the body's innate immune power to combat cancer. This approach has shown great promise in treating certain types of cancer and has fewer side effects than traditional single chemotherapy [10]. Overall, the search for new and better cancer drugs is a complex and ongoing process [11,12]. However, with continued research and innovation, we have hope for the future of cancer sufferers' treatment and quality of life.

**Scheme 1:**
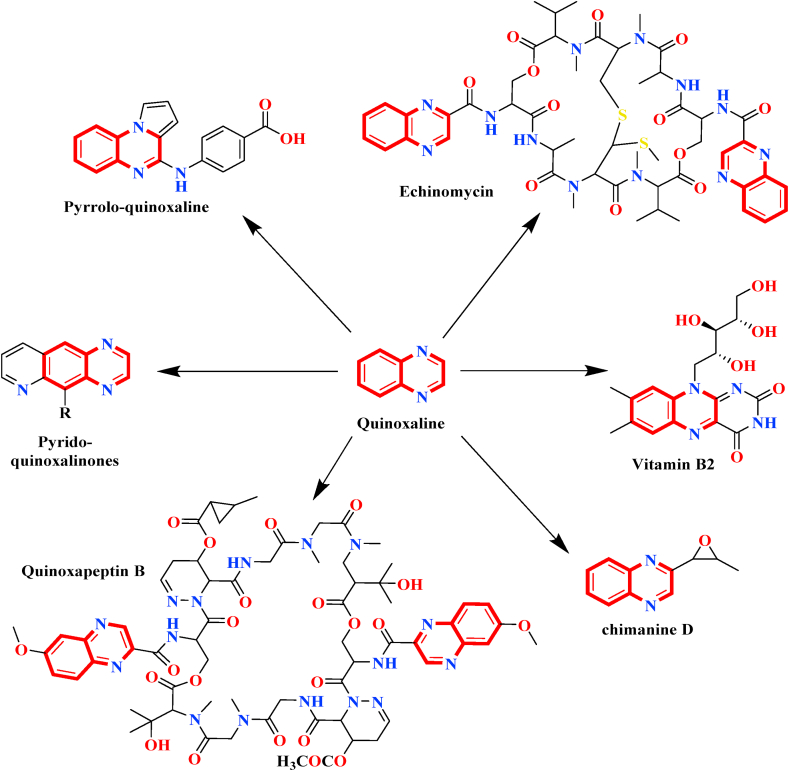
Quinoxaline as a scaffold for many natural products.

Triazolequinoxalin derivatives are a class of compounds that have been well studied for their various potential therapeutic applications (Scheme 1), including anti-cancer agents [13]. There are several reports on the synthesis strategies and biological application of triazolequinoxalin derivatives. For example, some studies have shown that these compounds exhibit anti-cancer activity encouraging apoptosis in specific cancer cells by targeting certain enzymes and receptors involved in cell growth and survival [14]. Other studies have investigated their potential as anti-microbial agents and found that they exhibit activity against several types of organisms from bacteria to fungi [15]. There are reports indicating that triazolequinoxalin derivative 2,3-bis(1,2,4-triazol-1-yl)quinoxaline displays anti-cancer effects on breast cancer cells through the initiation of cell cycle interruption and programmed cell death [16]. Nevertheless, further investigation is required to completely comprehend the potential of these compounds as therapeutic agents and to optimize their activity and selectivity for specific types of cancer or pathogens. Additionally, some triazolequinoxalin derivatives have demonstrated anti-inflammatory [17] and anti-HIV [18] properties, making them potentially useful in the treatment of other diseases as well. In view of the above-mentioned considerations and as a continuation of our research work [[19], [20], [21], [22], [23], [24], [25], [26], [27]], here we're offering a synthesis of triazolequinoxalin derivative. This study's particular objective is to confirm the structure of a synthetic molecule using spectroscopic techniques and XRD, whereas biological activity was predicted using molecular docking.

Computational chemistry has gained much attention in drug discovery due to its cost effectiveness, environment sustainablility, and zero waste of chemicals. In this study, DFT is one of the most accurate methods to determine the quantum parameters of atoms or molecules. Additionally, it has been implemented to calculate HOMO, LUMO, energy gap, hydrophilic and hydrophobic nature of triazolequinoxalin derivatives. Finally, the molecular docking study was performed to predict the anticancer potential of the titled compound by computing binding affinity and interactions with the active site residue of the target protein. Notably, further in-vitro and in-vivo experiments are crucial to improve the efficacy of the existing agents to cure cancer. As a result, effectively translating the promising relevance of such in-depth studies into cancer research has assisted in rethinking general principles regarding drug combinations and their mechanisms of action.

## 2 Result and discussion

### 2.1 Chemistry

**Scheme 2:**
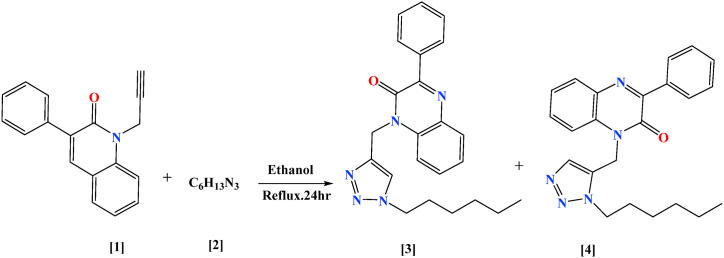
Reaction pathway for the synthesis of the title TZQ compounds (**3** and **4**).

The newly synthesized compound's structure (TZQ **4**) as shown in Scheme 2 has been identified using different spectroscopic findings of ^1^H, ^13^C NMR, and LC-MS, alongside C, H, and N analysis. The final structure is determined using the 1H NMR spectrum of the compound (TZQ **4**) as a distinctive example. This became readily apparent when the triple bond vanished in compound (**1**) without any difficulty. In addition to the triplet at *δ* 0.89 ppm which resulted from having three protons of the Methyl, then there are five methylene groups after the methyl have multiplet between *δ* 1.25–1.36 ppm was due to the six protons related to the three methylene groups after that quintet between *δ* 1.82–1.92 ppm related to the fourth methylene. Then the last methylene group in the terminal chain that connected to nitrogen of the triazole cycle exhibited triplet at *δ* 4.56 ppm. Furthermore, the most significant peak that differentiates the compounds (**3** and **4**) is the presence of a new proton of triazole ring at *δ* 7.64 ppm as a singlet due to its distance from the carbon chain. However, the CH group in compound (**3**) exhibits singlet at *δ* 7.95 ppm because of the near carbon chain. Finally, the multiplet between *δ* 7.36–8.33 ppm was attributed to the nine protons that remained in the aromatic groups. Moreover, for ^13^C NMR there is a notable increase in the peaks numbers related to the carbon chain that connected to the triazole cycle of the same final compound starting from 13.9 ppm for the terminal methyl group in addition of the peaks at *δ* 22.4, 26.2, 30.4, 31.2, 35.2 ppm for the five methylene group in the chain respectively (Supplementary file, Fig.S1 and Fig. S2). Ultimately, the mass spectrum becomes notable stability M+1 at *m/z* 388 that clearly affirmed the formation of the molecule (TZQ **4**) which is the same mass as the compound (TZQ **3**) (Supplementary file, Fig.S3).

### 2.2 Crystal structure of compound (4)

**Fig. 1:**
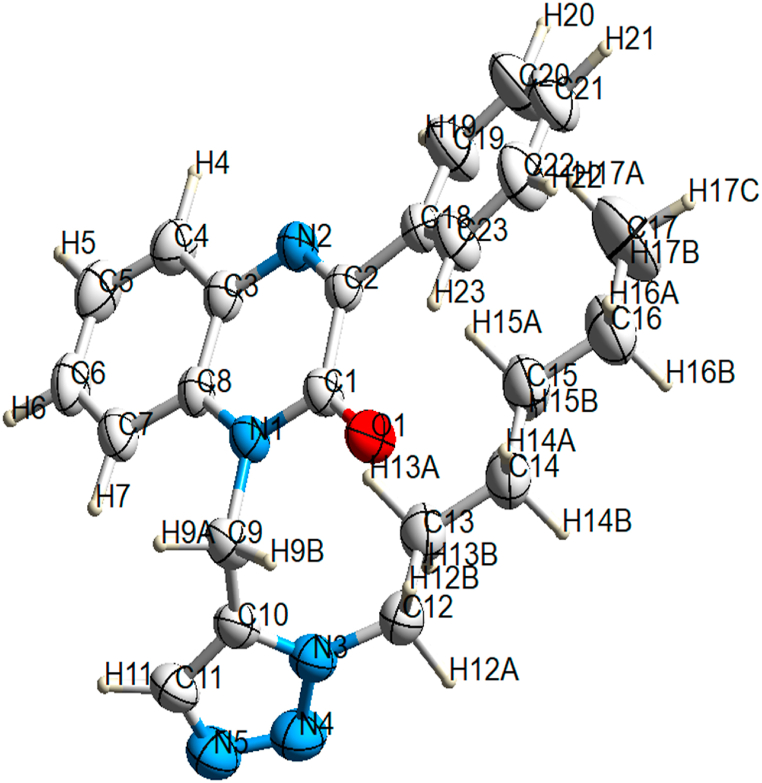
Molecular structure of compound (**4**) with anisotropic displacement ellipsoids drawn at 50 % probability level.

Compound (**4**) crystallizes within the monoclinic space group *C*2/*c,* housing a single molecule in the asymmetric unit as depicted in Fig. 1a. A comprehensive summary of crystal data and structure refinement particulars is presented in Table 1. The quinoxaline ring system is almost planar with an rmsd of 0.030 Å from planarity and atom N1 deviating most [0.052(1) Å]. The quinoxaline and phenyl ring (C18–C23) are inclined by 22.82(10)°. Meanwhile, the triazole ring maintain planar configuration with an rmsd of 0.004 Å and a maximal deviation of 0.005 Å for atom N3. The quinoxaline is turned nearly perpendicular to the triazole ring with a dihedral angle of 80.90 (9)° between both planes. The hexyl chain occurs in an extended form with torsion angles between −174.74(19) and 169.11(18)° along the C–C–C–C bonds (*antiperiplanar* conformations). One of the hexyl hydrogen atoms interacts with the pyrazinone ring (H13A⋅⋅⋅*Cg*2 = 2.81 Å, *Cg*2 is the centroid of the pyrazinone ring).

**Table 1:** Crystal data and structure refinement of compound (**4**).

Identification code	KC1009
Crystal system	monoclinic
Empirical formula	C_23_H_25_N_5_O
Temperature/K	293(2)
Formula weight	387.48
Space group	*C*2/*c*
α/°	90
β/°	93.904(4)
γ/°	90
a/Å	41.201(2)
b/Å	10.6339(6)
c/Å	9.4997(4)
Volume/Å^3^	4152.4(4)
Z	8
Crystal size/mm^3^	0.3 × 0.2 × 0.2
μ/mm^−1^	0.079
F(000)	1648
Reflections collected	21765
ρ_calc_g/cm^3^	1.24
2Θ range for data collection/°	4.848 to 52.744
Index ranges	−51 ≤ h ≤ 51, −13 ≤ k ≤ 13, −11 ≤ l ≤ 11
Goodness-of-fit on F^2^	1.03
Radiation	Mo Kα (λ = 0.71073)
Independent reflections	4236 [R_int_ = 0.0298, R_sigma_ = 0.0262]
Data/restraints/parameters	4236/0/268
Largest diff. peak/hole/e Å^−3^	0.18/-0.16
Final R indexes [I ≥ 2σ (I)]	R_1_ = 0.0507, w*R*_2_ = 0.1201
Final R indexes [all data]	R_1_ = 0.0753, w*R*_2_ = 0.1348

**Fig. 2:**
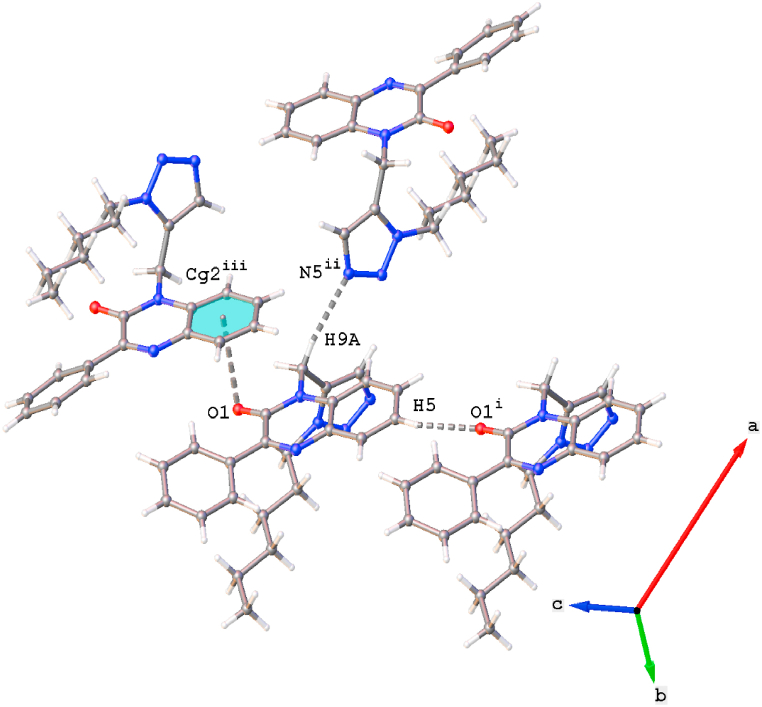
Partial crystal packing of compound (**4**) showing the C–H⋅⋅⋅O, C–H⋅⋅⋅N and C=O⋅⋅⋅π interactions. Symmetry codes: (i) *x*, *y*, −1+*z*; (ii) 3/2- *x*, −1/2+ *y*, 1/2- *z*, (iii) *x*, -*y*, ½+*z*.

The crystal packing of compound (**4**) is characterized by weak C5–H5⋅⋅⋅O1^i^ and C9–H9A⋅⋅⋅N5^ii^ interactions (Fig. 2, details in Table 2). Furthermore, a C=O⋅⋅⋅π interaction is observed between atom OP1 and the phenyl part of the quinoxaline ring [Fig. 2, O1⋅⋅⋅*Cg*3^iii^ = 3.4746(18) Å, *Cg*3 is the centroid of ring C3–C8, symmetry code: (iii) *x*, -*y*, ½+*z*].

**Table 2:** Hydrogen-bond geometry (Å, °) for compound (**4**).

D−H⋅⋅⋅A	D−H	H⋅⋅⋅A	D⋅⋅⋅A	D−H⋅⋅⋅A
C5–H5⋅⋅⋅O1^i^	0.93	2.39	3.283(3)	161
C9–H9A⋅⋅⋅N5^ii^	0.97	2.59	3.549(3)	171

Symmetry codes: (i) *x*, *y*, −1+*z*; (ii) 3/2- *x*, −1/2+ *y*, 1/2- *z.*

### 2.3 XRD and DFT calculations

**Fig. 3:**
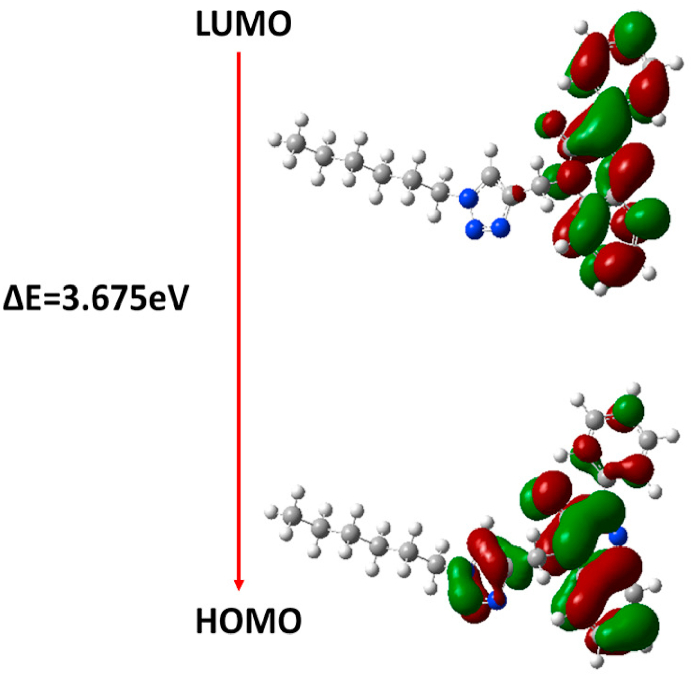
Frontier-molecular orbital HOMO-LUMO and energy gap of the title compound.

The monoclinic crystal system, space group *C*2/*c*, was determined for the titled quinoxaline derivative compound through a single crystal X-ray diffraction (XRD) study. The unit cell dimensions were measured as follows: a = 41.201(2) Å, b = 10.6339(6) Å, c = 9.4997(4) Å, β = 93.904(4), and Z = 8. An ORTEP diagram illustrating the title compound is provided in Fig. 1a. The structure of the compound was enhanced by the B3LYP/6-31+G(d, p) basis set via DFT calculations in the gas phase, employing the Gaussian 09 package [28]. The optimized molecular structure is shown in Fig. 1b. Additionally, the frontier molecular orbitals (HOMO-LUMO) are depicted in Fig. 3, and their energy gap, along with other global reactivity descriptors, are documented in Table 3.

**Table 3:** HOMO-LUMO and global reactivity descriptor values of the compound (**4**).

Parameters	Values (eV)
E_HOMO_	−5.864
E_LUMO_	−2.189
Ionization potential	5.864
Energy gap	3.675
Chemical hardness	1.837
Electron affinity	2.189
Chemical potential	−4.026
Global softness	0.272
Eletrophilicity	4.4117
Eletronegativity	4.026

### 2.4 Hirshfeld surface analysis of compound (4)

**Fig. 4:**
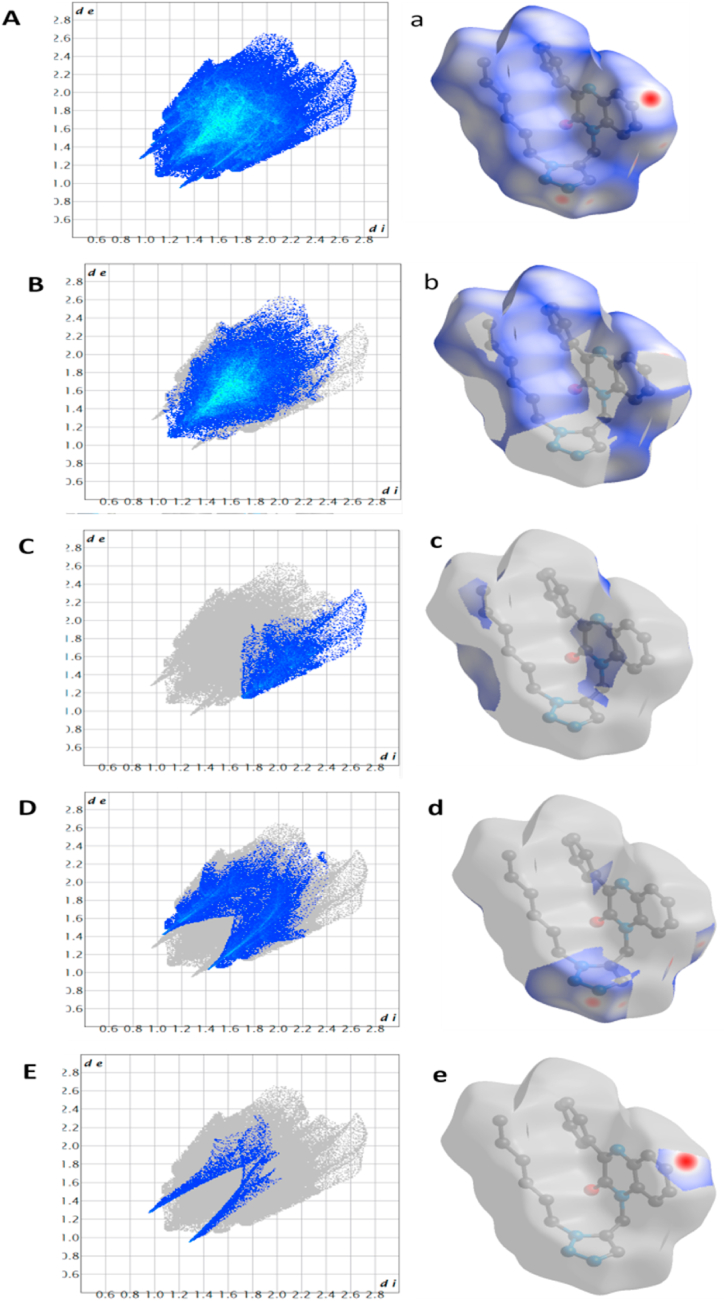
(**A**) Hirshfeld surface mapped over *d*_norm_, (**a**) Fingerprint plot of the compound from all the intermolecular contacts. (**B**), (**C**), (**D**) and (**E**) are the 2D fingerprint plots showing H–H, C–H, O–H, N–H contacts with the percentages contribution and (**b**), (**c**), (**d**) and (**e**) represent the associated *d*_norm_ Hirshfeld surfaces respectively.

**Fig. 5:**
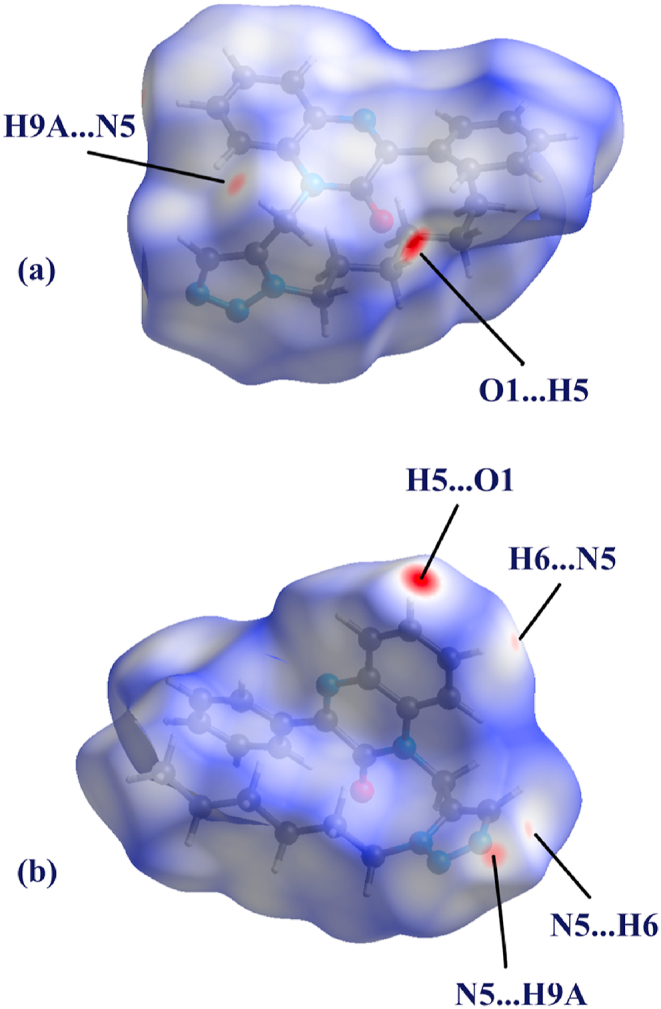
Two views (**a, b**) of the Hirshfeld surface mapped over *d*_norm_ for compound (**4**) over the range −0.2745 to 1.5767 a.u.

The crystal structure of the titled compound reveals enclosed intermolecular atomic contacts, which are assessed and visualized using Hirshfeld surfaces analysis. To perform this analysis and generate 2D fingerprint plots, we employed Crystal Explorer 17 software with the B3LYP function and 6-31G (d, p) basis set [29]. This analysis provides the explanation for the packing modes, intermolecular interaction and molecular surface properties of the crystal. The strengths and orientations of intermolecular interactions within the molecular crystal are depicted on Hirshfeld surfaces using the *d*_norm_ descriptor. This descriptor, derived from normalized contact distances (di and de) among the closest atoms both inside and outside the surface, offers valuable insights into these interactions. Compound (**4**) exhibits a resulting volume of 511.86 Å³ and a surface area of 404.23 Å^2^. The asphericity and globularity values for its shape are 0.09 and 0.766, respectively. Collectively, Fig. 4a visually demonstrates the combined contribution of all contacts to the overall Hirshfeld surface area. In this context, the dominant contribution emerges from H–H contacts at 57.7 % (Fig. 4b), whereas the other contacts' contributions come from C–H, N–H and O–H at 18.1, 17.7 and 2.7 % (Fig. 4c–e) respectively. Consequently, the contact analysis for this compound underscores that (H ….H) hydrogen bonds play significant impacts in explaining the molecular arrangement and the formation of the crystal packing. The highlighted red areas on the *d*_norm_ surface indicate contacts shorter than the combined van der Waals radii [30]. Five red spots on the Hirshfeld surface, color-coded by *d*_norm_, indicate the presence of strong C–H⋯O hydrogen bond interactions, characterized by their brevity compared to van der Waals radii. Conversely, blue regions correspond to longer contacts with positive *d*_norm_ values. Utilizing two-dimensional (2D) fingerprint plots, visualization depicts the relative contribution of different intermolecular interactions in relation to the molecule's overall dimension (Fig. 5a and b).

### 2.5 Shape index, curvedness, and electrostatic potential map

**Fig. 6:**
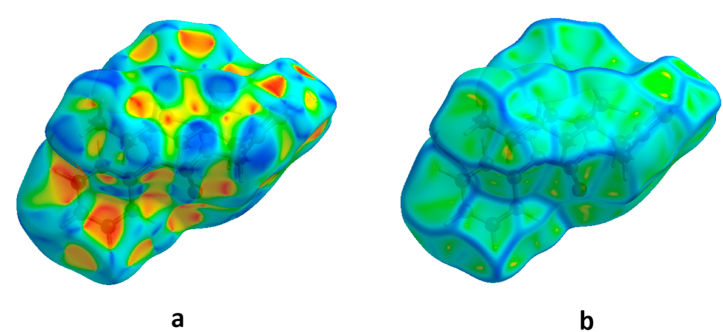
Hirshfeld surface mapped over (**a**) shape index and (**b**) curvedness.

Within our crystal structure, we have thoroughly examined the intermolecular interactions present in the asymmetric unit through the use of 2D fingerprint maps. These maps illustrate the proportions of various types of intermolecular interactions that occupy different areas. Additionally, we have employed 3D Hirshfeld surfaces, shape index, and curvedness analysis on the molecule. The d_norm_ surfaces are color-coded to signify distinct regions [31]. Specifically, blue denotes positive electrostatic potential, while red highlights areas with negative electrostatic potential. The d_norm_ value calculates the ratio of normalized interaction on the surface at any given point to the closest interior (di) and exterior (de) atom, considering the atom's van der Waals radius. Shape index alterations are most easily discerned on the surface (Fig. 6a), with the curvedness map showcasing a considerable green region, segmented by dark blue curves (Fig. 6b). There are few flat regions, observed in the surface map that indicates planar stacking between molecules. Fig. 5a and b illustrate the compound's electrostatic potential on the generated Hirshfeld surface. The electronegative areas (hydrogen bond acceptors) are depicted in red, while electropositive regions (hydrogen bond donors) are indicated in blue [32].

### 2.6 Energy frameworks

**Fig. 7:**
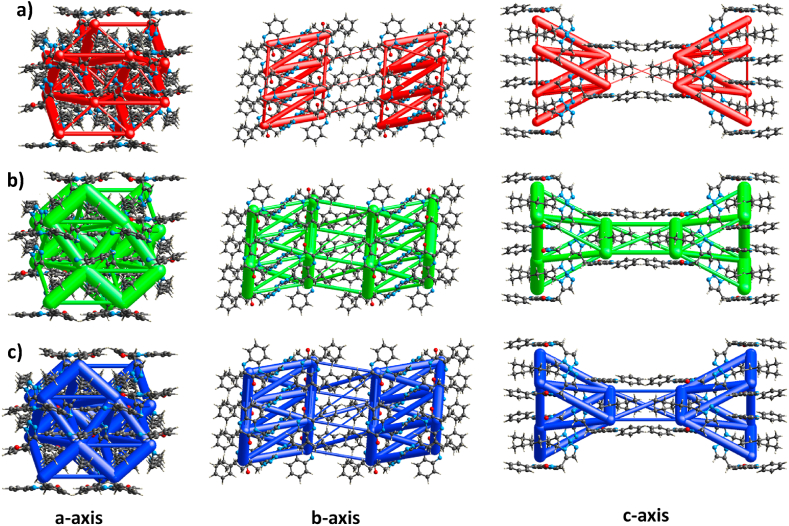
The pictorial illustration of **a)** electrostatic coulomb interaction energy depicted in red, **b)** dispersion energy depicted in green, **c)** total interaction energy depicted in blue along different axes of the title compound.

We can determine the type of interaction in terms of energies that construct the supramolecular structure in the crystal by quantifying the energies from the energy framework [33]. These computations were performed using the Crystal Explorer program at the B3LYP/6-31G (d, p) level. All interaction energies were determined within a 3.8 Å cluster centered on an individual molecule of the TZQ **4**. The energy framework for B3LYP/6-31G (d, p) was established using the following scale factors: k_ele = 1.057, k_pol = 0.740, k_disp = 0.871, k_rep = 0.618 [34]. The outcomes for electrostatic, dispersion, polarization, and repulsion energies are detailed in Table 4. The calculated electrostatic, dispersion, polarization, repulsion and total energies were −68.3, −234.7, −20.8, 119.8 and −217.8 kJ/mol respectively. According to these results, dispersion energies are dominant over other interaction energies. Fig. 7a-c illustrates representations of the compound's electrostatic coulomb interaction energy (red, Fig. 7a), dispersion energy (green, Fig. 7b), and total interaction energy (blue, Fig. 7c) along the a, b, and c axes. To get a clear overview of these energies by controlling the size of the cylinder, an overall scale factor is applied [35]. Few interactions were ignored under certain threshold energy to make the figures less packed.

**Table 4:** Various interaction energies including electrostatic, dispersion, polarization and repulsion energies of the molecular pairs (kJ/mol), using B3LYP/6-31G(d,p) as electron density model.

N	Symop	R	E_ele	E_pol	E_dis	E_rep	E_tot
2	x, -y, z+1/2	7.52	−4.3	−1.5	−49.1	25.0	−33.0
2	-x+1/2, y+1/2, -z+1/2	10.97	−22.4	−7.0	−16.3	17.3	−32.3
1	-x, -y, -z	13.24	−1.0	−0.2	−7.9	0.0	−8.0
2	x, y, z	9.50	−8.8	−2.1	−15.8	17.3	−13.8
1	-x+1/2, -y+1/2, -z	11.67	−15.4	−4.6	−16.4	15.2	−24.6
2	x, -y, z+1/2	6.76	−6.0	−1.7	−59.1	24.5	−44.0
1	-x+1/2, -y+1/2, -z	9.67	−7.3	−3.1	−28.5	10.9	−28.1
1	-x, -y, -z	12.82	−2.3	−0.1	−16.3	0.0	−16.7
1	-x, y, -z+1/2	11.26	−0.7	−0.3	−18.5	9.6	−11.1
1	-x, y, -z+1/2	14.17	−0.1	−0.2	−6.8	0.0	−6.2

### 2.7 Docking simulation

The human inducible phospho-fructokinase bisphosphatase isoform 3, PFK3, plays a crucial role in regulating the intracellular fructose-2,6-bisphosphate level due to relative high kinase activity. Thus, inhibition of glycolysis or kinase activity emerges as a promising approach for cancer therapy.

**Fig. 8:**
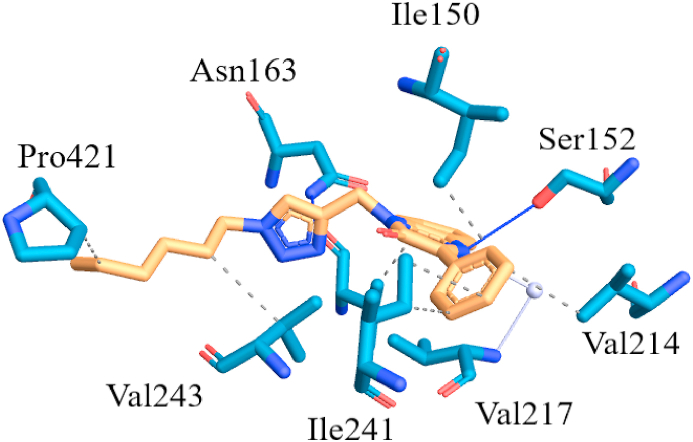
The pictorial representation of the compound (**4**), binded inside the cavity of pocket of PFKFB3 Kinase.

Among the reported PFK3 inhibitors, quinoxalines showed potential inhibition [36,37]. Taking this into consideration, the crystal structure of PFK3 protein (PDB Code: **6HVH**) has been used for molecular docking study. Fig. 8 depicted the binding mode of the compound (**4**). Docking results indicated the strong binding affinity of compound (**4**) with docking score of −8.5 kcal/mol towards target protein. The detailed overview of the binding mode reveals the formation of multiple hydrophilic and hydrophobic interactions with the crucial residues of the protein. The nitrogen of the triazole ring implicated in the interaction of hydrogen bond with Asn 163 at a distance of 2.9 Å. While the second hydrogen bond is observed between the nitrogen atom of quinoxaline moiety with Ser 152 at a distance of 3.00 Å. These protein-ligand interactions were further stabilized by hydrophobic interaction with key residues Ile 50, Val 159, Val 214, Ile 241, Val 243 and Pro 421. Consequently, the docking results shows significant binding interaction of compound (**4**) indicating its potential as a promising anti-cancer molecule in the future.

## 3 Conclusions

In this investigation, we subjected compound (**4**), a derivative of quinoxaline, to characterization through LC-MS, NMR spectra, and X-ray diffraction methods. The X-ray crystallography unveiled that compound (**4**) arranges itself within the monoclinic crystal system with the *C*2/*c* space group. To delve into its molecular geometry and electronic attributes, we conducted DFT calculations employing the 6–311G++(d, p) basis set, revealing a commendable concurrence between experimental and structural parameters. Scrutinizing the frontier molecular orbitals, encompassing HOMO and LUMO, as well as their energy gaps, we noted charge transfer interactions from the ground to the first excited state. The stability of the compound is upheld by both intermolecular and intramolecular C–H⋯O hydrogen bonds and C–H⋯N interactions. Through an analysis of the electrostatic potential, we gained insights into the intramolecular interactions within the compound.

We conducted Hirshfeld surface investigations to determine the contribution of intermolecular interactions, and the results showed that H–H contacts account for the majority of the total contribution, accounting for 57.7 % of the total. Moreover, we performed an energy framework analysis based on various interaction energies, with the electrostatic energy emerging as the most influential among all interactions. In addition to its structural properties, we assessed the compound's potential as an anticancer agent through molecular docking studies. The outcome indicated significant binding affinity towards the target protein, with prominent hydrophilic, hydrophobic, and *π-π* stacking interactions at the active site. Consequently, compound (**4**) emerges as a promising lead compound for the development of potential anticancer agents. In conclusion, the comprehensive characterization of compound (**4**) sheds light on its molecular and structural properties, reinforcing its potential as a lead candidate for future anti-cancer drug development. Finally, to enhance the current agents, methods, and combinations of drugs for the use of cancer therapy, further laboratory investigations, preclinical studies, and well-designed clinical trials are needed.

## 4 Materials and methods

### 4.1 Experimental section

The reagents, chemicals and solvents needed to obtain the title compound (**4**) were supplied by Fisher Chemicals and Sigma Aldrich Chemical Co without any additional purification. The advancement of the reaction was checked on TLC plates, and the spots were made visible using UV light at wavelengths of 254 nm. Chemiline (microcontroller-based melting point/boiling point-Cl725 apparatus with a digital thermometer) was used to gauge melting and boiling points. The 1H and 13C NMR spectrums were acquired at ambient temperature on a VNMRS-300 Agilent-NMR spectrophotometer. Mass spectra have been captured on the API 3200 LC/MS/MS mass spectrometer using electrospray ionization (ESI) in positive mode. The findings of the elemental analysis are accurate to within 0.5 % of the theoretical value.

#### 4.1.1 Synthesis procedure for preparation of compounds (TZQ 3 and 4)

The 0.5 g (0.0022 mmol) of dipolarophile 3-phenyl-1-(prop-2-yn-1-yl) quinoxalin-2(1*H*)-one (**1**), was added to (1.3 equiv) of 1-azidohexan (**2**) in absolute ethanol (20 ml). Under reflux, the reaction mixture was heated and maintained for 24 h, followed by TLC monitoring [Ethyl acetate/Hexane (1:9 (v/v)]. The solvent was evaporated under a vacuum. Following the concentration at reduced pressure, the residue was purified using column chromatography with silica gel and an eluent consisting of a 1:1:9 (v/v) mixture of ethyl acetate and hexane. The resulting solid product was then crystallized using ethanol to give both final compounds (**3**) as powder and (**4**) as off-white crystal with (30 % and 60 % yield) respectively [38].

##### 4.1.1.1 1-((1-Hexyl-1H-1,2,3-triazol-4-yl)methyl)-3-phenylquinoxalin-2(1H)-one(3)

Eluent (hexane/ethyl acetate (90/10 %); Yield: = 60 %; F(°C) = 97–99; NMR ^1^H (300 MHz, CDCl_3_)*δ* ppm: 0.85–0.89 (t, 3H, *J* = 6 Hz, CH_3_); 1.25–1.36 (m, 6H, CH_2_); 1.82–1.92 (quin, 2H, CH_2_); 4.27–4.32 (t, 2H, *J* = 6 Hz, N–N–CH_2_); 5.63 (S, 2H, N–CH_2_); 7.95 (s, H, CH_Traizol_); 7.37–8.35 (m, 9H_arom_); NMR^13^C (75 MHz, CDCl_3_) *δ* ppm: 13.8 (CH_3_); 22.3, 26.1, 30.0, 31.0, 38.4 (CH_2_); 50.4 (N–CH_2_); 114.7, 123.6 (CH_arom_), 124.0_Traizol_, 128.1, 129.4, 130.4, 130.4, 130.7 (CH_arom_); 132.4, 133.35, 135.9, 142.4, 153.8 (Cq); 154.6 (C=O); LC-MS *m/z* 388 [M+]. Anal. Calcd. for C_23_H_25_N_5_O(387): C, 71.29; H, 6.50; N, 18.07. Found: C, 71.22; H, 6.47; N, 18.01 %. (Supplementary file, Fig.S4 and Fig. S5).

##### 4.1.1.2 1-((1-Hexyl-1H-1,2,3-triazol-5-yl)methyl)-3-phenylquinoxalin-2(1H)-one (4)

Eluent (hexane/ethyl acetate (90/10 %); Yield: = 30 %; F(°C) = 125–127; NMR^1^H (300 MHz, CDCl_3_)*δ* ppm: 0.87-0.91 (t, 3H, CH_3_, *J* = 6_Hz_); 1.29–1.33 (m, 6H, CH_2_); 1.84–1.94 (quin, 2H, CH_2_); 4.50–4.64 (t, 2H, *J* = 6 Hz, N–C**H**_**2**_–CH_2_); 5.63 (s, 2H, N–C**H**_**2**_–C); 7.64 (s, H, CH_Traizol_); 7.36–8.33 (m, 9H_arom_); NMR^13^C (75 MHz, CDCl_3_) *δ* ppm:13.9 (CH_3_); 22.4, 26.2, 30.4, 31.2, 35.2 (CH_2_); 48.7 (N–CH_2_); 113.3, 124.5 (C-H_arom_), 128.2_Traizol_, 129.5, 130.7, 130.7, 131.1, 131.2 (CH_arom_); 131.8, 133.5, 133.6, 135.5, 153.8 (Cq); 154.1 (C=O); LC-MS *m/z* 388 [M+]. Anal. Calcd. for C_23_H_25_N_5_O(387): C, 71.29; H, 6.50; N, 18.07. Found: C, 71.22; H, 6.47; N, 18.01 %. (Supplementary file, Fig.S1, S2 and Fig. S3).

### 4.2 Crystal structure determination of compound (4)

MoK radiation was used to capture X-ray intensity data at 293(2) K on an Agilent SuperNova diffractometer with an Eos CCD detector in order to produce a single crystal of compound (**4**). Intensity data integration, correction for Lorentz and polarization effects, and empirical absorption correction were performed on the pictures using CrysAlisPRO. With the aid of Olex 2 [39], the structure was solved using Intrinsic Phasing by ShelXT [40] and then refined by ShelXL [37] using full-matrix least-squares minimization on F2. In the riding mode, the H atoms were arranged in an ideal manner. The riding mode refined the molecules of hydrogen with isotropic temperature factors fixed at 1.2 times Ueq of the parent atoms (1.5 times Ueq for CH_3_), whereas non-hydrogen atoms were refined anisotropically. Table 1 provides a summary of crystal data, data collection, and structural refinement details.

### 4.3 Geometry optimization

The molecular structure of **TZQ (4)** was subjected to optimization by the DFT approach with B3LYP and a 6-31+G(d,p) basis set, utilizing the Gaussian 09W software package [28]. The initial structure was constructed using Gauss View of molecular visualization software. The chemical reactivity of the **TZQ (4)** molecule was elucidated through global reactivity descriptors, MEP, and HOMO-LUMO energies.

### 4.4 Docking simulation

In order to explore the anticancer potential of compound (**4**), docking studies were conducted on the target protein PFKFB3 kinase using the PDB Code: **6HVH** [41]. Prior to initiating the docking procedure, the protein was prepared by removing co-crystal ligands, cofactors, and solvent molecules, while other issues in protein preparation like missing atoms, charge neutralization, and addition of polar hydrogen bonds were fixed using protein preparation module in MOE [42]. The compound (**4**) was structurally modeled in a 3D conformation using MOE's builder module and then subjected to minimization through the MMFF94x force field [43]. For the purpose of docking investigations, the induced fit docking protocol was employed, using a Triangular matcher for placement and London dG as the scoring function. Thirty conformations were generated and saved in mdb format.

## Data availability statement

Data associated with this study has been deposited at Cambridge Crystallographic Data Centre under the accession number CCDC: 225879**5**.

## CRediT authorship contribution statement

**Nadeem Abad:** Conceptualization, Data curation, Formal analysis, Investigation, Methodology, Validation, Visualization, Writing – original draft, Software, Writing – review & editing. **Fares Hezam Al-Ostoot:** Conceptualization, Data curation, Formal analysis, Investigation, Methodology, Resources, Software, Supervision, Validation, Visualization, Project administration, Writing - original draft, Writing - review & editing. **Sajda Ashraf:** Conceptualization, Data curation, Formal analysis, Methodology, Software, Validation, Writing – original draft. **Karim Chkirate:** Conceptualization, Investigation, Methodology, Project administration, Supervision, Validation, Visualization, Writing – original draft. **MajedS. Aljohani:** Conceptualization, Data curation, Investigation, Validation, Visualization, Writing – review & editing. **HussamY. Alharbi:** Conceptualization, Data curation, Methodology, Resources, Validation, Visualization, Writing – review & editing. **Shafeek Buhlak:** Conceptualization, Formal analysis, Resources, Software, Supervision, Validation, Writing – review & editing. **Mohamed El Hafi:** Formal analysis, Project administration, Resources, Supervision, Validation, Visualization, Writing – review & editing. **Luc Van Meervelt:** Conceptualization, Data curation, Formal analysis, Methodology, Project administration, Software, Supervision, Validation, Writing – review & editing. **Basheer M. Al-Maswari:** Conceptualization, Formal analysis, Methodology, Supervision, Validation, Writing – review & editing. **El Mokhtar Essassi:** Conceptualization, Investigation, Project administration, Resources, Supervision, Validation, Visualization, Writing – review & editing. **Youssef Ramli:** Data curation, Formal analysis, Project administration, Resources, Supervision, Validation, Visualization, Writing – review & editing.

## Declaration of competing interest

The authors declare that they have no known competing financial interests or personal relationships that could have appeared to influence the work reported in this paper.
