# Supplementary material for: Synthesis, crystal structure, DFT, Hirshfeld surface analysis, energy frameworks and in-Silico drug-targeting PFKFB3 kinase of novel triazolequinoxalin derivative (TZQ) as a therapeutic Strategy against cancer
Source: Heliyon. 2023 Oct 20;9(11):e21312. doi: 10.1016/j.heliyon.2023.e21312 (PMC10618769; doi:10.1016/j.heliyon.2023.e21312)
Supplement: Multimedia component 1 [file mmc1.docx]

**Supplemntary File:**


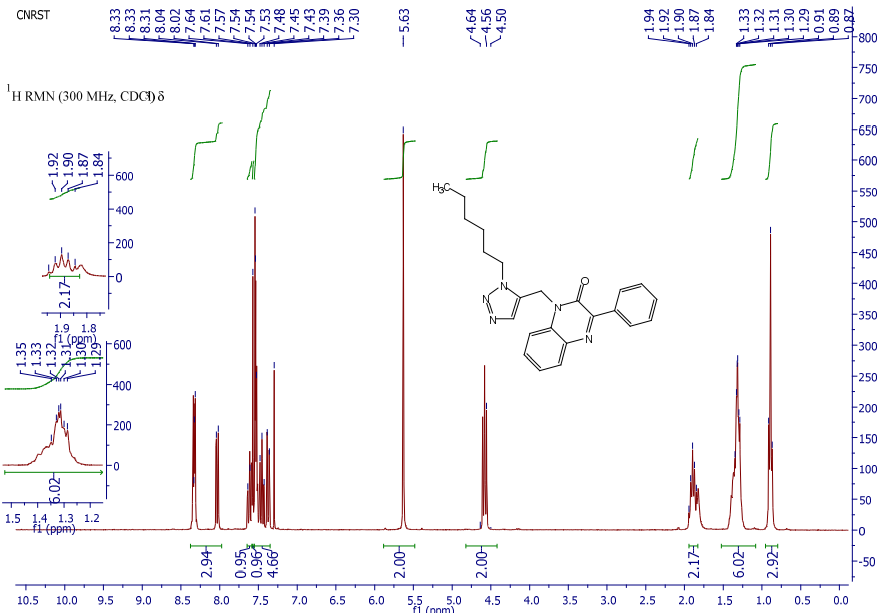


**Fig.S1.** ^1^HNMR spectrum of TZQ compound **(4).**


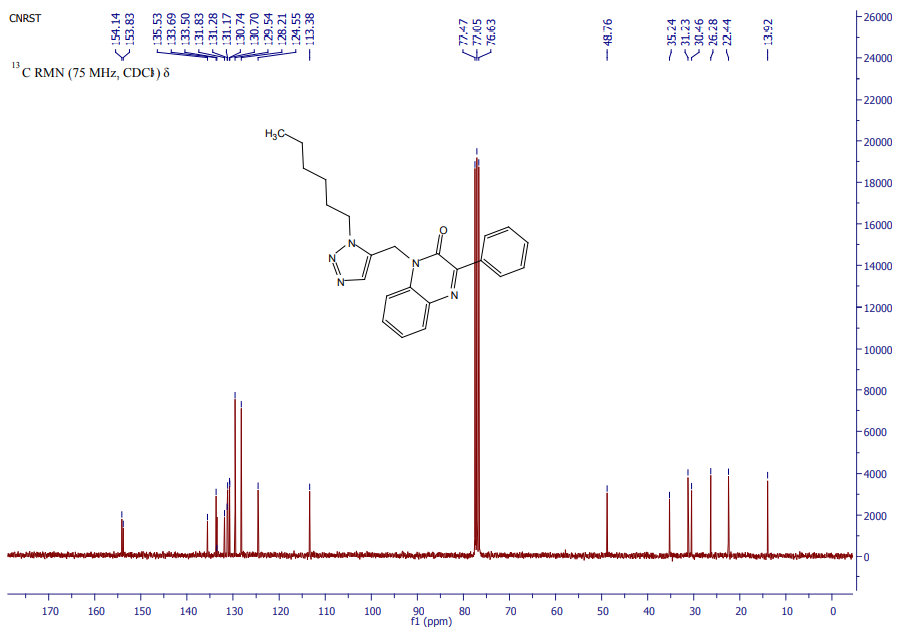


**Fig.S2.** ^13^CNMR spectrum of TZQ compound **(4).**


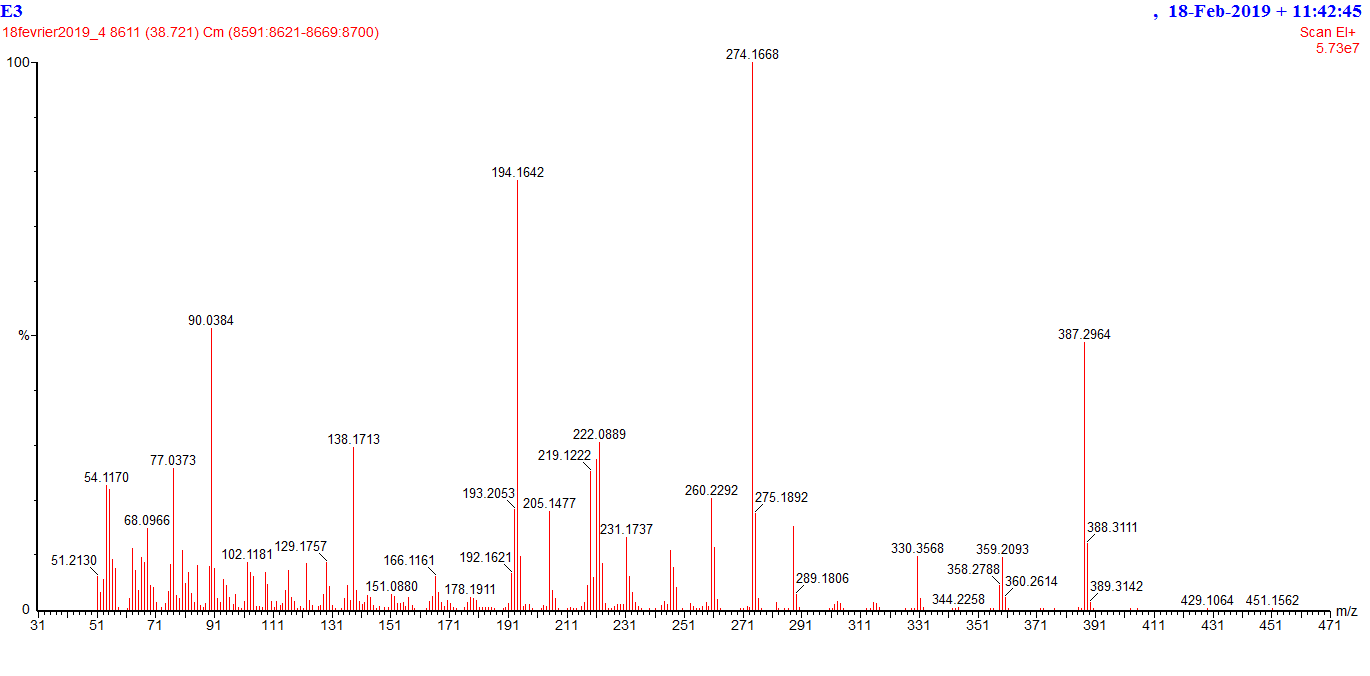


**Fig.S3.** Mass spectrum of TZQ compound (**4**).


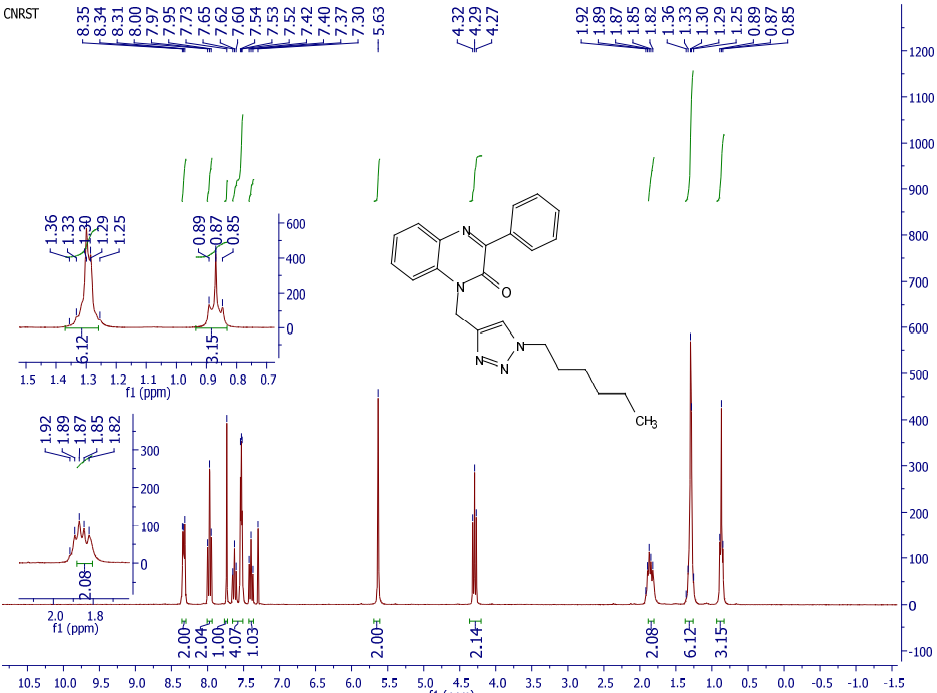


**Fig.S4.** ^1^HNMR spectrum of TZQ compound **(3)**


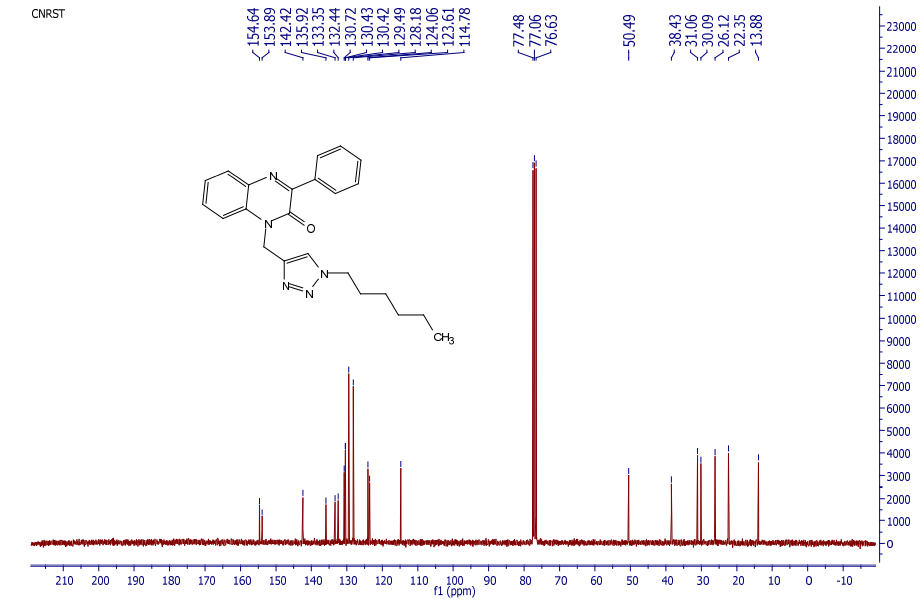


**Fig.S5.** ^13^CNMR spectrum of TZQ compound **(3)**

**Table S1: Bond Lengths for Compound (4).**

| **Atom** | **Atom** | **Length/Å** |  | **Atom** | **Atom** | **Length/Å** |
| --- | --- | --- | --- | --- | --- | --- |
| O1 | C1 | 1.225(2) |  | C5 | C6 | 1.379(3) |
| N1 | C1 | 1.384(2) |  | C6 | C7 | 1.373(3) |
| N1 | C8 | 1.395(2) |  | C7 | C8 | 1.397(2) |
| N1 | C9 | 1.481(2) |  | C9 | C10 | 1.496(3) |
| C1 | C2 | 1.487(2) |  | C10 | C11 | 1.369(3) |
| N2 | C2 | 1.295(2) |  | C12 | C13 | 1.516(3) |
| N2 | C3 | 1.385(2) |  | C13 | C14 | 1.519(3) |
| C2 | C18 | 1.489(2) |  | C14 | C15 | 1.512(3) |
| N3 | N4 | 1.350(2) |  | C15 | C16 | 1.516(3) |
| N3 | C10 | 1.349(3) |  | C16 | C17 | 1.508(4) |
| N3 | C12 | 1.464(2) |  | C18 | C19 | 1.383(3) |
| C3 | C4 | 1.399(2) |  | C18 | C23 | 1.378(3) |
| C3 | C8 | 1.396(2) |  | C19 | C20 | 1.377(3) |
| N4 | N5 | 1.313(3) |  | C20 | C21 | 1.369(4) |
| C4 | C5 | 1.372(3) |  | C21 | C22 | 1.354(3) |
| N5 | C11 | 1.349(3) |  | C22 | C23 | 1.387(3) |

**Table S2 Bond Angles for Compound (4).**

| **Atom** | **Atom** | **Atom** | **Angle/˚** |  | **Atom** | **Atom** | **Atom** | **Angle/˚** |
| --- | --- | --- | --- | --- | --- | --- | --- | --- |
| C1 | N1 | C8 | 122.43(14) |  | N1 | C8 | C3 | 117.47(15) |
| C1 | N1 | C9 | 117.64(14) |  | N1 | C8 | C7 | 123.35(16) |
| C8 | N1 | C9 | 119.92(14) |  | C3 | C8 | C7 | 119.18(16) |
| O1 | C1 | N1 | 120.95(16) |  | N1 | C9 | C10 | 113.80(15) |
| O1 | C1 | C2 | 123.57(16) |  | N3 | C10 | C9 | 125.96(17) |
| N1 | C1 | C2 | 115.41(14) |  | N3 | C10 | C11 | 103.91(19) |
| C2 | N2 | C3 | 119.96(15) |  | C11 | C10 | C9 | 130.1(2) |
| C1 | C2 | C18 | 120.86(15) |  | N5 | C11 | C10 | 109.5(2) |
| N2 | C2 | C1 | 122.35(16) |  | N3 | C12 | C13 | 112.25(17) |
| N2 | C2 | C18 | 116.78(16) |  | C12 | C13 | C14 | 112.37(18) |
| N4 | N3 | C12 | 119.49(18) |  | C15 | C14 | C13 | 113.16(18) |
| C10 | N3 | N4 | 110.85(17) |  | C14 | C15 | C16 | 114.1(2) |
| C10 | N3 | C12 | 129.54(17) |  | C17 | C16 | C15 | 113.2(2) |
| N2 | C3 | C4 | 118.06(17) |  | C19 | C18 | C2 | 118.32(17) |
| N2 | C3 | C8 | 122.14(15) |  | C23 | C18 | C2 | 124.16(17) |
| C8 | C3 | C4 | 119.77(17) |  | C23 | C18 | C19 | 117.39(18) |
| N5 | N4 | N3 | 107.25(18) |  | C20 | C19 | C18 | 121.4(2) |
| C5 | C4 | C3 | 120.47(19) |  | C21 | C20 | C19 | 120.3(2) |
| N4 | N5 | C11 | 108.48(18) |  | C22 | C21 | C20 | 119.2(2) |
| C4 | C5 | C6 | 119.23(19) |  | C21 | C22 | C23 | 120.8(2) |
| C7 | C6 | C5 | 121.79(19) |  | C18 | C23 | C22 | 120.9(2) |
| C6 | C7 | C8 | 119.52(19) |  |  |  |  |  |

**Table S3 Torsion Angles for Compound (4).**

| **A** | **B** | **C** | **D** | **Angle/˚** |  | **A** | **B** | **C** | **D** | **Angle/˚** |
| --- | --- | --- | --- | --- | --- | --- | --- | --- | --- | --- |
| O1 | C1 | C2 | N2 | 174.21(18) |  | C4 | C3 | C8 | N1 | 177.71(16) |
| O1 | C1 | C2 | C18 | -4.9(3) |  | C4 | C3 | C8 | C7 | -2.0(3) |
| N1 | C1 | C2 | N2 | -2.7(2) |  | C4 | C5 | C6 | C7 | -1.4(3) |
| N1 | C1 | C2 | C18 | 178.17(15) |  | C5 | C6 | C7 | C8 | -0.3(3) |
| N1 | C9 | C10 | N3 | 67.6(2) |  | C6 | C7 | C8 | N1 | -177.69(17) |
| N1 | C9 | C10 | C11 | -115.4(2) |  | C6 | C7 | C8 | C3 | 2.0(3) |
| C1 | N1 | C8 | C3 | 4.8(2) |  | C8 | N1 | C1 | O1 | -178.68(17) |
| C1 | N1 | C8 | C7 | -175.56(17) |  | C8 | N1 | C1 | C2 | -1.7(2) |
| C1 | N1 | C9 | C10 | -112.73(18) |  | C8 | N1 | C9 | C10 | 67.8(2) |
| C1 | C2 | C18 | C19 | 160.1(2) |  | C8 | C3 | C4 | C5 | 0.3(3) |
| C1 | C2 | C18 | C23 | -24.2(3) |  | C9 | N1 | C1 | O1 | 1.9(3) |
| N2 | C2 | C18 | C19 | -19.1(3) |  | C9 | N1 | C1 | C2 | 178.88(15) |
| N2 | C2 | C18 | C23 | 156.61(19) |  | C9 | N1 | C8 | C3 | -175.77(15) |
| N2 | C3 | C4 | C5 | -178.15(18) |  | C9 | N1 | C8 | C7 | 3.9(3) |
| N2 | C3 | C8 | N1 | -3.9(2) |  | C9 | C10 | C11 | N5 | -178.41(19) |
| N2 | C3 | C8 | C7 | 176.40(16) |  | C10 | N3 | N4 | N5 | -0.7(2) |
| C2 | N2 | C3 | C4 | 178.15(17) |  | C10 | N3 | C12 | C13 | -88.3(3) |
| C2 | N2 | C3 | C8 | -0.2(3) |  | C12 | N3 | N4 | N5 | -177.21(17) |
| C2 | C18 | C19 | C20 | 175.5(2) |  | C12 | N3 | C10 | C9 | -5.3(3) |
| C2 | C18 | C23 | C22 | -174.6(2) |  | C12 | N3 | C10 | C11 | 177.00(19) |
| N3 | N4 | N5 | C11 | 0.1(2) |  | C12 | C13 | C14 | C15 | -174.74(19) |
| N3 | C10 | C11 | N5 | -0.8(2) |  | C13 | C14 | C15 | C16 | -179.7(2) |
| N3 | C12 | C13 | C14 | 169.11(18) |  | C14 | C15 | C16 | C17 | -179.2(2) |
| C3 | N2 | C2 | C1 | 3.6(3) |  | C18 | C19 | C20 | C21 | -0.7(5) |
| C3 | N2 | C2 | C18 | -177.22(15) |  | C19 | C18 | C23 | C22 | 1.1(3) |
| C3 | C4 | C5 | C6 | 1.4(3) |  | C19 | C20 | C21 | C22 | 1.1(5) |
| N4 | N3 | C10 | C9 | 178.64(17) |  | C20 | C21 | C22 | C23 | -0.5(4) |
| N4 | N3 | C10 | C11 | 0.9(2) |  | C21 | C22 | C23 | C18 | -0.7(4) |
| N4 | N3 | C12 | C13 | 87.5(2) |  | C23 | C18 | C19 | C20 | -0.5(4) |
| N4 | N5 | C11 | C10 | 0.4(3) |  |  |  |  |  |  |
